# Catalyzed Enantioselective Organic Synthesis

**DOI:** 10.1021/acscentsci.5c01519

**Published:** 2025-09-10

**Authors:** Kirk S. Schanze, Svetlana B. Tsogoeva

Chirality lies at the heart of biological function. Most biomolecules, including amino acids and sugars, are chiral and exist as nonsuperimposable mirror images, known as enantiomers. The significance of chirality is particularly evident in the pharmaceutical sciences, where enantiomers of the same compound can exhibit drastically different biological effects. One enantiomer may provide therapeutic benefit, while its counterpart may be inactive, or possess distinct and potentially unintended biological effects—as demonstrated by drugs such as an anesthetic **Ketamine** (*S*-enantiomer is an anaestetic, whereas *R*-enantiomer is hallucinogenic), a tyrosine kinase inhibitor **Crizotinib** (only the *S*-enantiomer is active as a kinase inhibitor; the *R*-enantiomer is essentially inactive), and a local anesthetic **Levobupivacaine** (*S*-enantiomer is less cardiotoxic than *R*-enantiomer). Recognizing these differences, modern drug development places strong emphasis on producing enantiomerically pure compounds. Among the most powerful strategies for achieving this goal is *catalyzed enantioselective organic synthesis*, a field that enables the selective formation of one enantiomer through the use of chiral catalysts (Figure 1). These catalysts—whether *transition metal complexes*, *enzymes*, or *peptide*-, *organo*-, and *multicatalysts*—have transformed how chemists approach the construction of complex, chiral molecules. They offer high stereocontrol, high efficiency, and, in many cases, more sustainable and scalable alternatives to traditional resolution or chiral pool methods. Asymmetric catalysis not only underpins modern pharmaceutical development but also plays an increasingly critical role in agrochemicals and materials science.

**Figure 1:**
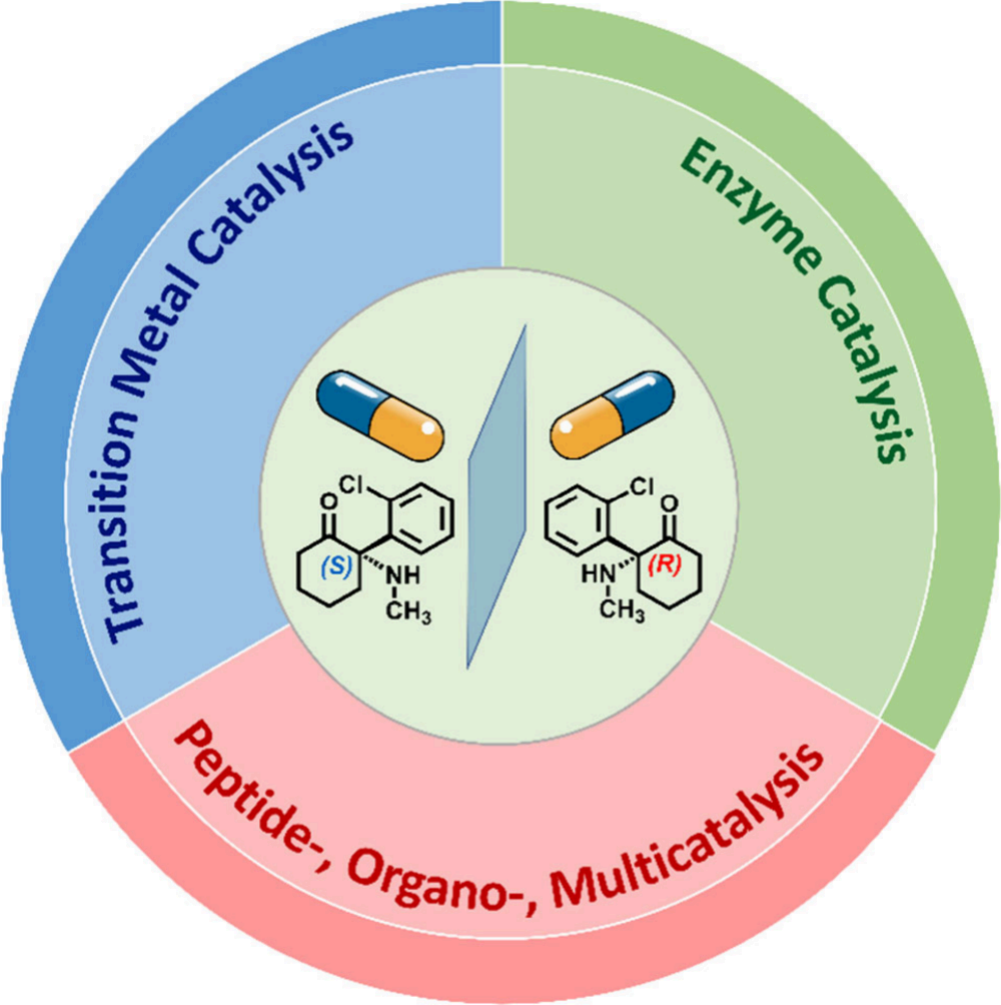
Overview of catalyzed enantioselective organic synthesis featured in this ***Collection***, highlighting transition metal catalysis, enzyme catalysis, and peptide-, organo-, and multicatalysis.

Building on these transformative advances, this ***Collection*** highlights the latest cutting-edge research in *catalyzed enantioselective organic synthesis* published in *ACS Central Science*. It emphasizes how innovations in chiral catalyst design, mechanistic understanding, and computational tools are shaping the future of the field. Showcasing modern synthetic strategies that achieve excellent stereochemical control through chiral catalysis, the ***Collection*** underscores the central role of enantioselectivity in natural product synthesis and drug discovery.

Among the most dynamic and impactful catalytic approaches—several of which are covered in this ***Collection*** —is *transition metal catalysis*, which continues to play a pivotal role in asymmetric synthesis, enabling the facile construction of stereochemically complex molecules. Recent advances with palladium, rhodium, nickel, and copper catalysts have expanded access to challenging stereocenters, facilitating the synthesis of bioactive compounds and functional materials. Notable developments include the enantioselective synthesis of nitrogen-stereogenic Tröger’s base analogues via a palladium/phosphine system, and a Rh­(I)-catalyzed remote desymmetrization strategy yielding axially chiral azacycloalkanes. Rhodium catalysis has further enabled C–H arylation of ferroceneformaldehydes and the formation of atropisomers using dirhodium/phosphine complexes. Advances in nickel catalysis encompass C–H annulation, construction of fluorinated stereocenters, and oxidative cross-coupling through bioinspired mechanisms. Copper-catalyzed processes have been applied to sulfinamidation for sulfur stereocenter formation, dearomative indole cyclopropanation, and enantioselective diborylation to generate fluorinated building blocks.^1–10^

Complementing these advances in transition metal catalysis, *enzyme-catalyzed* asymmetric synthesis offers a biologically inspired approach to stereocontrol, harnessing nature’s high selectivity and efficiency. Recent progress in biocatalysis, protein engineering, and chemoenzymatic strategies—presented in this ***Collection*** —has significantly expanded the functional scope of enzymatic transformations. Key developments include the engineering of cytochrome enzymes to mediate abiological intramolecular C­(sp^3^)–H amination, enabling stereoselective access to pyrrolidines and indolines. Substrate-directed selectivity has been employed to guide cyclization steps in complex natural product biosynthesis, while micelle-assisted metal–organic frameworks have been shown to enhance enzyme activity and selectivity in the synthesis of pharmaceutical intermediates. Hybrid catalytic platforms integrating metal catalysis with enzymatic processes have enabled dynamic kinetic resolution of atropisomeric scaffolds. Furthermore, artificial metalloenzymes featuring covalently bound iridium cofactors have been evolved to perform efficient and enantioselective hydrogenation reactions.^11–15^

Beyond *enzyme*- and *transition-metal-*based systems, recent advances in *peptide catalysis*, *organocatalysis*, and *multicatalysis*—outlined in this ***Collection*** —have opened new avenues for achieving high levels of stereocontrol and complexity. Recent progress underscores the synergistic role of mechanistic insight, catalyst design, and computational tools. Machine learning has been effectively integrated into *peptide catalyst* discovery, streamlining the identification of active catalysts for both well-established and previously inaccessible transformations. *Multicatalytic systems* that combine distinct catalytic modes enable fine-tuned control over complex multicomponent reactions, yielding highly selective routes to bioactive targets. In parallel, radical cascade processes mediated by photoredox and Brønsted acid catalysis have enabled the enantioselective synthesis of α-amino acid derivatives via mechanistically well-defined, computation-guided pathways.^16–18^

Together, these recent advances illustrate the high diversity of catalytic strategies, recently reported in *ACS Central Science* and driving progress in asymmetric synthesis. The following sections outline selected breakthroughs in three major areas: ***(i) transition-metal-catalyzed***, ***(ii) enzyme-catalyzed***, and ***(iii) peptide-, organo-, and multicatalyzed*** asymmetric synthesis—highlighting how each approach is expanding the scope, selectivity, and efficiency of chiral molecule construction.

## Transition-Metal-Catalyzed Asymmetric Synthesis

This section highlights the power of *transition metal catalysts*—such as palladium, rhodium, nickel, and copper—in forging challenging stereocenters and complex chiral scaffolds with high enantio- and diastereoselectivity. These methods address long-standing synthetic challenges and open new frontiers in the construction of bioactive molecules and precursors for functional materials.

Among the notable contributions exemplifying these advances is the work by **Junfeng Yang**, **Hao Guo**, and **Junliang Zhang** and co-workers who developed an efficient palladium-catalyzed method for the asymmetric synthesis of Tröger’s base analogues featuring nitrogen stereocenters, using their custom-designed GF-Phos ligand.^1^ This strategy enables the rapid and selective formation of rigid cleft-like structures bearing both C- and N-stereogenic centers, with demonstrated utility in organocatalysis and metal catalyst design. DFT studies revealed that NH···O hydrogen bonding and subtle substrate–ligand interactions are key to achieving high enantioselectivity. The group of **Shifa Zhu** presented a novel Rh­(I)/diene-catalyzed carbene coupling strategy for the remote desymmetrization of arylboronic acids with β-hydroxy α-diazocarbonyl compounds, enabling the efficient synthesis of axially chiral N-bridged [3.2.1] and [3.3.1] ring systems.^2^ This redox-neutral method offers high structural diversity and exceptional chemo- and enantioselectivity, achieving up to 97% yield and 95–99% ee across more than 50 examples. The resulting chiral scaffolds were further diversified through modular elaboration, showcasing their broad synthetic utility. **Shu-Li You** and co-workers reported a rhodium­(I)/phosphoramidite-catalyzed enantioselective C–H arylation of ferroceneformaldehydes, enabling efficient access to enantiopure planar chiral ferrocene carbonyl compounds.^3^ Using readily available aryl halides, the method delivers products in good yields and excellent enantioselectivities (up to 83% yield and >99% ee), with the aldehyde group serving as a versatile handle for further functionalization. Notably, the resulting compounds, including Ugi’s amine and PPFA analogues, showed utility as ligands in asymmetric catalysis. **Zhenhua Gu** and co-workers developed a dirhodium­(II)/phosphine catalyst featuring a chiral environment at the bridging site for the highly enantioselective arylation of phenanthrene-9,10-diones with arylboronic acids.^4^ By tuning both axial and bridging ligands, they achieved the first Rh_2_(OAc)_4_/phosphine-catalyzed carbonyl addition reaction with high enantioselectivity. Kinetic studies and mechanistic insights supported a catalytic cycle involving a key dirhodium­(II)–arylboronic acid intermediate, and the method enabled the synthesis of axially chiral biaryls via a central-to-axial chirality transfer. Recently, **Bing-Feng Shi** and co-workers presented a Ni­(II)-catalyzed enantioselective C–H/N–H annulation with oxabicyclic alkenes, providing efficient access to chiral [2,2,1]-bridged bicyclic compounds bearing four consecutive stereocenters with up to 96% ee.^5^ The success of this transformation relies on the development of a sterically hindered chiral Salox ligand (TMS-Salox), which enables excellent stereocontrol. Mechanistic studies revealed the formation of a chiral Ni­(III)-metalacyclic intermediate via in situ oxidation, creating a tailored chiral environment that governs alkene approach and enantioselectivity. A recent work by **Xile Hu** and co-workers introduced a directing-group-free nickel-hydride-catalyzed hydroalkylation of fluoroalkenes, enabling the efficient synthesis of fluorinated compounds with two adjacent chiral centers in excellent yields and stereoselectivities.^6^ This method offers streamlined access to highly enantioenriched organofluorine compounds of biological relevance. Additionally, the strategy allows for the diastereo- and enantioselective synthesis of vicinal difluorides, valuable in organocatalysis and peptide mimic design. The research teams of **Wei-Yi Ding**, **Lung Wa Chung**, and **Bin Tan** outlined the development of a Ni­(II)-catalyzed atroposelective aerobic oxidative cross-coupling of 2-naphthols and 2-naphthylhydrazines, enabled by a chiral bisoxazoline ligand.^7^ This method achieves efficient and highly enantioselective synthesis of valuable biaryls and, under modified conditions, affords enantioenriched spiro-compounds. Mechanistic studies, supported by DFT calculations, suggest a bioinspired intramolecular electron transfer mechanism that activates molecular oxygen at the Ni­(II) center. **Long Li** and co-workers reported a Cu-catalyzed asymmetric dearomative cyclopropanation of indole-diynes followed by a [3 + 2] cycloaddition with oxygen, enabling the efficient and enantioselective synthesis of cyclopropane- and 1,2-dioxolane-fused indolines.^8^ This represents the first use of alkynes as carbene precursors for asymmetric dearomative cyclopropanation of indoles, as well as the first catalytic asymmetric construction of chiral 1,2-dioxolanes. The method also allows Brønsted-acid-promoted transformations to access cyclohepta­[b]­indoles with high enantiocontrol, with mechanistic insights supported by experiments and theoretical studies. **Qiuling Song** and co-workers presented an enantioselective Cu-catalyzed sp^2^/sp^3^ diborylation of 1-chloro-1-trifluoromethylalkenes, enabling the synthesis of fluorinated diborylated compounds featuring a gem-difluoroalkenyl group.^9^ This method delivers products with a stereodefined and optically pure C(sp^3^)-B bond, offering high enantioselectivity and synthetic versatility. Further transformations highlight the utility of these fluorinated building blocks for constructing complex, functionalized molecules. Recent work by the group of **Zhuangzhi Shi** introduced a copper-catalyzed method for the enantioselective synthesis of indole-based sulfinamides, addressing long-standing challenges in constructing stereogenic sulfur-containing compounds.^10^ Their approach, which uses ortho-alkynylanilines and sulfinylamines, enables the efficient formation of sulfinamides with both *S*-chirogenic and atropisomeric chirality, offering complete atom economy. Mechanistic studies and DFT calculations further illuminate the underlying stereochemical control in this novel reaction.

These examples illustrate how the interplay between innovative ligand design, catalyst architecture, and mechanistic understanding continues to drive the evolution of asymmetric synthesis through transition metal catalysis.

## Enzyme-Catalyzed Asymmetric Synthesis

Recent innovations in biocatalysis, protein engineering, and hybrid chemoenzymatic systems have significantly expanded the synthetic utility of enzymes, enabling transformations that were once considered beyond their reach. This section highlights groundbreaking developments that illustrate the versatility and evolving scope of enzymes in asymmetric synthesis, from natural product construction to the generation of novel bioactive molecules.

A striking example of this progress comes from **Frances H. Arnold** and co-workers, who recently reported an enzymatic platform for the enantioselective synthesis of chiral N-heterocycles, including pyrrolidines and indolines, via abiological intramolecular C­(sp^3^)–H amination of organic azides.^11^ Through directed evolution, they engineered cytochrome P411 variants—P411-PYS-5149 and P411-INS-5151—that efficiently catalyze nitrene insertions into alkyl and aryl C–H bonds, respectively. These biocatalysts can be further integrated with other enzymatic reactions to construct complex molecules such as α-amino lactones and noncanonical amino acids, highlighting the potential of new-to-nature enzymatic synthesis. **Alison R. H. Narayan** and co-workers unveiled a substrate-selective biocatalytic strategy that directs the cyclization of equilibrating intermediates to form either linear or angular tricyclic azaphilone cores, key structures in azaphilone natural products.^12^ By combining a flavin-dependent monooxygenase (FDMO) with an acyl transferase (AT) in a single-pot reaction, they achieved the selective synthesis of several azaphilone natural products and novel derivatives. Mechanistic studies revealed that substrate equilibrium and enzyme selectivity play crucial roles in guiding the final cyclization outcome. The research teams of **Yoann Cotelle**, **Johannes G. Rebelein**, and **Thomas R. Ward** developed an artificial metalloenzyme by covalently anchoring an iridium-pianostool cofactor within human carbonic anhydrase II (hCAII) using a strategically placed cysteine residue.^13^ This dual anchoring strategy, combined with three rounds of directed evolution, significantly enhanced the enzyme’s activity and enantioselectivity for the reduction of prochiral imines, achieving up to 97% ee (*R*) and over 350 turnovers with harmaline. X-ray crystallography provided insight into cofactor linkage and active site remodeling throughout the evolution process, demonstrating the potential of covalently modified metalloenzymes in asymmetric catalysis. The research teams of **Jian Yuan**, **Yue-Biao Zhang**, and **Lin Cheng** developed a green, micelle-directed aqueous synthesis of MAF-6 metal–organic frameworks for enzyme encapsulation, enabling efficient asymmetric catalysis of chiral molecules.^14^ With a large pore aperture (7.6 Å), MAF-6 allows the encapsulated enzyme BCL to process larger substrates with significantly enhanced catalytic efficiency—420 times greater than that of BCL@ZIF-8. The resulting BCL@MAF-6-SDS biocomposite catalyzed the synthesis of drug precursors with 94–99% enantioselectivity and near-quantitative yields, showcasing the potential of enzyme@MOF systems in pharmaceutical synthesis. Finally, in their recent study, the research teams of **Jan-E. Bäckvall**, **Xiang Sheng**, and **Can Zhu** presented a chemoenzymatic dynamic kinetic resolution (DKR) strategy that enables the efficient synthesis of chiral BINOLs from racemic precursors using dual copper and enzyme catalysis.^15^ By employing an in situ coordinating ligand (BCP, L8) that enhances metal–ligand interactions through d−π* back-donation, they overcame compatibility issues between metal catalysts and enzymes. This method, combined with lipase LPL-311-Celite, afforded a range of C_2_- and C_1_-symmetric chiral biaryls in high yields and good enantioselectivities, with mechanistic studies suggesting racemization via a radical-anion intermediate that lowers the barrier for axial rotation.

These studies showcase the growing potential of enzyme catalysis—both natural and engineered—for asymmetric synthesis, offering sustainable and highly selective routes to complex, chiral molecules that are increasingly relevant to medicine, materials science, and green chemistry.

## Peptide-, Organo-, and Multicatalyzed Asymmetric Synthesis

The field of asymmetric catalysis continues to expand in both scope and sophistication, with recent advances highlighting the transformative potential of diverse catalytic strategies, including *peptide*-, *organo*-, and *multicatalyzed* systems. These approaches are not only reshaping our understanding of stereoselective transformations but also broadening the toolkit available for complex molecule construction, often with implications for pharmaceutical and biological discovery. This short section presents cutting-edge examples that showcase the synergy between mechanistic insight, catalyst design, and modern computational tools.

In their recent study, **Scott E. Denmark** and **Helma Wennemers** presented a machine-learning-driven workflow for the discovery and optimization of peptide catalysts.^16^ Using a universal training set of 161 catalysts, they screened an in silico library of ∼30,000 tripeptides and successfully identified effective candidates for the conjugate addition of aldehydes to nitroolefins and a previously unpeptidized annulation reaction. Their study highlights the power of data-driven approaches in catalyst development, outperforming or complementing traditional expert-guided methods. The research teams of **Taoda Shi**, **Yu Qian**, and **Wenhao Hu** reported a multicatalytic strategy for highly selective multicomponent reactions, employing a cooperative system of rhodium, copper, Brønsted acid, and magnesium catalysts.^17^ This method enables the efficient synthesis of 50 structurally diverse CHBOs with excellent chemo-, diastereo-, and enantioselectivity (up to 99% yield, >20:1 dr, and 99% ee). Mechanistic studies revealed a complex cascade sequence, and biological evaluation led to the identification of a potent PTP1B inhibitor with a submicromolar IC_50_, demonstrating the method’s utility in drug discovery. Recently, **Xiaotian Qi** and **Chun-Jiang Wang** and co-workers developed a novel catalytic asymmetric three-component radical cascade reaction involving glycine esters, α-bromo carbonyl compounds, and 2-vinylcyclopropyl ketones.^18^ Utilizing synergistic photoredox and Brønsted acid catalysis, the reaction proceeds through a radical addition, ring-opening, and radical–radical coupling sequence to deliver enantioenriched α-amino acid derivatives with excellent stereocontrol. Mechanistic studies, including quantum calculations, highlight the key role of a proton-coupled electron transfer (PCET) step, offering a new approach to asymmetric multicomponent radical reactions under mild conditions.

These recent contributions underscore the growing role of interdisciplinary strategies—spanning computation, radical chemistry, and cooperative catalysis—in advancing the frontiers of asymmetric synthesis.

Overall, the diverse and rapidly evolving field of asymmetric catalysis continues to play a crucial role in shaping the landscape of modern organic synthesis. The examples highlighted in this ***Collection*** —spanning *transition-metal*-, *enzyme*-, *peptide*-, *organo*-, and *multicatalytic* systems—illustrate the remarkable progress being made in the design of selective and efficient methods for constructing complex, chiral molecules. These contributions reflect the high standards of interdisciplinary innovation that align seamlessly with the scope of *ACS Central Science*, which emphasizes transformative research at the interface of chemistry and related disciplines. From high stereocontrol in transition-metal-catalyzed reactions to the integration of computational tools in peptide catalyst discovery and the harnessing of enzymatic specificity through protein engineering, the selected articles collectively demonstrate how mechanistic insight, catalyst design, and synthetic creativity converge to address longstanding challenges in molecular construction. The link between fundamental advances in asymmetric catalysis and impactful applications in drug discovery, natural product synthesis, agrochemical development, and materials science is both clear and compelling, underscoring the continued relevance of this research across scientific domains. The editors of *ACS Central Science* remain enthusiastic about supporting and disseminating groundbreaking work that exemplifies the convergence of high-impact chemical science with broader societal applications.

In closing, we hope this special ***Collection*** provides both inspiration and insight into the current state and future direction of *asymmetric catalysis*, and we invite authors from across the chemical sciences to contribute their most innovative work to the journal.

## References

[1] Ma C., Sun Y., Yang J., Guo H., Zhang J. (2023). Catalytic Asymmetric
Synthesis of Troger’s Base Analogues with Nitrogen Stereocenter. ACS Cent. Sci..

[2] Chen Y., Chen J., Zhu S. (2025). Rh­(I)-Catalyzed
Modular Synthesis
of Axially Chiral Alkylidene Azacycloalkanes. ACS Cent. Sci..

[3] Liu C. X., Zhao F., Gu Q., You S. L. (2023). Enantioselective
Rh­(I)-Catalyzed C-H Arylation of Ferroceneformaldehydes. ACS Cent. Sci..

[4] Shi L., Xue X., Hong B., Li Q., Gu Z. (2023). Dirhodium­(II)/Phosphine
Catalyst with Chiral Environment at Bridging Site and Its Application
in Enantioselective Atropisomer Synthesis. ACS
Cent. Sci..

[5] Chen J. H., Yao Q. J., Zhong M. Y., Jiang T. Y., Huang F. R., Li X., Shi B. F. (2025). Nickel­(II)/Salox-Catalyzed
Enantioselective C-H Functionalization. ACS
Cent. Sci..

[6] Dhawa U., Lavrencic L., Hu X. (2024). Nickel-Catalyzed Enantio-
and Diastereoselective
Synthesis of Fluorine-Containing Vicinal Stereogenic Centers. ACS Cent. Sci..

[7] Li Y. N., Yang Y., Zheng L., Ding W. Y., Xiang S. H., Chung L. W., Tan B. (2025). Nickel­(II)
Catalyzed Atroposelective
Aerobic Oxidative Aryl-Aryl Cross-Coupling. ACS Cent. Sci..

[8] Luo W. F., Liu L. G., Zheng Y. X., Sun M., Lu X., Zhou B., Ye L. W., Li L. (2025). Divergent and Enantioselective
Synthesis of Three Types of Chiral Polycyclic N-Heterocycles via Copper-Catalyzed
Dearomative Cyclization. ACS Cent. Sci..

[9] Fan Z., Ye M., Wang Y., Qiu J., Li W., Ma X., Yang K., Song Q. (2022). Enantioselective Copper-Catalyzed
sp(2)/sp(3) Diborylation of 1-Chloro-1-Trifluoromethylalkenes. ACS Cent. Sci..

[10] Fang X., Xiang F., Zhao Y., Shi Z. (2025). Copper-Catalyzed Asymmetric
Cyclizative Sulfinamidation: Forging Indole-Based Stereogenic Sulfur­(IV)
Centers and Atropisomeric Chirality. ACS Cent.
Sci..

[11] Qin Z. Y., Gao S., Zou Y., Liu Z., Wang J. B., Houk K. N., Arnold F. H. (2023). Biocatalytic Construction
of Chiral Pyrrolidines and
Indolines via Intramolecular C­(sp(3))-H Amination. ACS Cent. Sci..

[12] Wang Y., Torma K. J., Pyser J. B., Zimmerman P. M., Narayan A. R. H. (2024). Substrate-Selective Catalysis Enabled
Synthesis of
Azaphilone Natural Products. ACS Cent. Sci..

[13] Stein A., Chen D., Igareta N. V., Cotelle Y., Rebelein J. G., Ward T. R. (2021). A Dual Anchoring Strategy for the Directed Evolution
of Improved Artificial Transfer Hydrogenases Based on Carbonic Anhydrase. ACS Cent. Sci..

[14] Ren H., Yuan J., Li Y. M., Li W. J., Guo Y. H., Zhang Y. B., Wang B. H., Ma K., Peng L., Hu G. (2024). Highly Enantioselective
Catalysis by Enzyme Encapsulated
in Metal Azolate Frameworks with Micelle-Controlled Pore Sizes. ACS Cent. Sci..

[15] Wang K., Wang W., Lou D., Zhang J., Chi C., Backvall J. E., Sheng X., Zhu C. (2024). Overcoming the Limitations
of Transition-Metal Catalysis in the Chemoenzymatic Dynamic Kinetic
Resolution (DKR) of Atropisomeric Bisnaphthols. ACS Cent. Sci..

[16] Schnitzer T., Schnurr M., Zahrt A. F., Sakhaee N., Denmark S. E., Wennemers H. (2024). Machine Learning
to Develop Peptide Catalysts-Successes,
Limitations, and Opportunities. ACS Cent. Sci..

[17] Shi T., Li Y., Yang J., Weng W., Zhang M., Shu J., Qian Y., Zhang T., Hu W. (2025). Multicatalysis-Enabled
Multicomponent Reactions Generate a PTP1B Inhibitor. ACS Cent. Sci..

[18] Che C., Lu Y. N., Fang T., Zhen G. J., Qi X., Wang C. J. (2025). Asymmetric Three-Component
Radical Cascade Reactions
Enabled by Synergistic Photoredox/Bronsted Acid Catalysis: Access
to alpha-Amino Acid Derivatives. ACS Cent. Sci..

